# Does surgically resected small‐cell lung cancer without lymph node involvement benefit from prophylactic cranial irradiation?

**DOI:** 10.1111/1759-7714.13381

**Published:** 2020-03-06

**Authors:** Yuqing Lou, Runbo Zhong, Jianlin Xu, Rong Qiao, Jiajun Teng, Yanwei Zhang, Xueyan Zhang, Tianqing Chu, Hua Zhong, Baohui Han

**Affiliations:** ^1^ Department of Respiration, Shanghai Chest Hospital Shanghai Jiao Tong University Shanghai China

**Keywords:** Brain metastases, prophylactic cranial irradiation, resected, small cell lung cancer

## Abstract

**Background:**

It has previously been demonstrated that surgically resected small‐cell lung cancer (SCLC) patients could benefit from prophylactic cranial irradiation (PCI). However, PCI in patients without lymph node involvement remains controversial. This study includes a larger sample size to evaluate the benefit of PCI therapy in this specific population.

**Methods:**

The records of surgically resected SCLC patients without lymph node involvement (N0M0) in Shanghai Chest Hospital were retrospectively reviewed.

**Results:**

Between January 2006 and May 2017, a total of 146 cases of surgically resected SCLC without lymph node involvement were included. A total of 46 patients received PCI therapy and 100 patients received no therapy. During the observation period, 12.0% (12/100) of the patients who did not receive PCI therapy developed brain metastases while 10.9% (5/46) of patients who received PCI therapy developed brain metastases. With regard to time to recurrence, no significant difference was observed among the groups (*P* = 0.798). Moreover, there was no significant difference in either the overall survival benefit (hazard ratio [HR] = 0.84, 95% confidence interval [CI]: 0.49–1.45, *P* = 0.532) or disease‐free survival rate (HR = 0.95, 95% CI: 0.52–1.75, *P* = 0.864).

**Conclusions:**

The evidence obtained does not support PCI therapy in the management of surgically resected SCLC with no lymph node involvement.

**Key points:**

Prophylactic cranial irradiation (PCI) remains controversial for resected small‐cell lung cancer (SCLC) without lymph node involvement. In this study, the results indicated that PCI does not reduce the risk of cerebral recurrence of resected p‐T1‐2N0M0 SCLC.

This is the largest sample size study focused on PCI in resected p‐T1‐2N0M0 SCLC. Future revised versions of the guidelines should address this issue.

## Introduction

Small cell lung cancer (SCLC) comprises a minor percentage of lung cancer diagnoses, although it is characterized by the early onset of widespread metastases.^1^, ^2^ While there have been remarkable enhancements in the management options of non‐small cell lung cancer (NSCLC), the treatment and ultimately the prognosis of SCLC have not altered. For 30 years, etoposide plus platinum have continued to be the first‐line therapy among this population. Recently, atezolizumab plus etoposide and platinum has been approved as the first‐line choice.^3^ As most chemotherapy agents have difficulty penetrating the blood‐brain barrier, nearly half of all SCLC patients encounter a brain relapse.^4^

Prophylactic cranial irradiation (PCI) diminishes the rate of brain metastases while improving survival for limited stage SCLC patients.^5^, ^6^ Previously, we also demonstrated that patients with resected SCLC could benefit from PCI. However, its use on patients without lymph node involvement remains controversial.^7^ The National Comprehensive Cancer Network (NCCN) and European Society for Medical Oncology (ESMO) guidelines endorse PCI therapy in patients fitting this diagnosis based on evidence in limited stage SCLC patients.^8^ Several retrospective reports have demonstrated the benefits of PCI therapy in prolonging overall survival (OS) and reducing the probability of brain metastases for completely resected SCLC with p‐N1‐N2M0,^9^, ^10^ but there is less evidence for its use in patients without lymph node involvement. Péchoux *et al*. published a review recommending PCI therapy for p‐T1‐2N0M0 SCLC,^11^ yet the conclusion was debated by Knisely *et al*. among this subset of patients.^12^ On the basis of our earlier work,^7^ we observed a larger sample size of p‐T1‐2N0M0 SCLC patients to assess the benefit of PCI therapy.

## Methods

### Patients

We retrospectively reviewed cases of SCLC with lung resection performed at Shanghai Chest Hospital from January 2006 to May 2017. The inclusion criteria were: (i) p‐SCLC patients who had undergone surgical resection as the primary treatment for their tumor and (ii) patients without lymph node involvement. The exclusion criteria were: (i) patients who had microscopically positive resections; (ii) wedge resections; (iii) died within 30 days post‐surgery; and (iv) those lost to follow‐up. All patients underwent preoperative tests, including chest CT scans, brain MRI, and abdominal CT scans or ultrasonography, or positron emission tomography (PET)/CT scans. The Institutional Review Board of Shanghai Chest Hospital (Shanghai, China) approved this study. As a retrospective study, informed consent was not required.

### Statistical analysis

Disease‐free survival (DFS) as well as OS are presented as the median with two‐sided 95% confidence interval (CIs), computed by the Kaplan‐Meier method. Differences between groups were found using the log‐rank test. Hazard ratios and their 95% CIs were analyzed with the use of a Cox proportional‐hazards analysis. The cumulative incidence of brain metastases were assessed via the Kaplan‐Meier method while comparisons between cohorts used the log‐rank test. Statistical significance was defined as *P* < 0.05. SPSS software, version 22 (SPSS Inc., Chicago, IL, USA) was used for all statistical analyses.

## Results

Between January 2006 and May 2017, a total of 146 surgically resected SCLC patients without lymph node involvement were finally included in this study (19 cases who were lost to follow‐up were excluded). Among this sample, 46 cases underwent PCI therapy while 100 patients did not (Table 1). The median follow‐up time was 27.5 months. Overall, 63 (43.2%) patients incurred a recurrence while 52 (35.6%) patients died following surgery. The pattern of recurrence is demonstrated in Table SS1. Of the 146 patients with resected SCLC, 92 (63.0%) patients underwent preoperative biopsies. A total of 25 (17.1%) patients were preoperatively diagnosed with SCLC, while 49 (33.6%) patients were diagnosed with other types of cancer (including unclassified carcinoma). A total of 89 (61.0%) patients received a PET scan before surgery. Of the total 146 SCLC patients, 91 (62.3%) had pure SCLC and 55 (37.7%) had combined SCLC according to histopathological analyses of the surgical specimens. Large cell lung cancer (*n* = 23) was the most frequent codiagnosis, followed by squamous carcinoma (*n* = 16), adenocarcinoma (*n* = 10), and adenosquamous carcinoma (*n* = 6) (Table 1).

**Table 1:** Demographic data of all patients

Characteristic	PCI therapy (*n* = 46)	No PCI therapy (*n* = 100)	*P*‐value
Median age (range)	63 (42–76)	63 (35–82)	
Gender			
Male	40 (87.0%)	88 (88.0%)	0.859
Female	6 (13.0%)	12 (12.0%)	
Smoking status			
Smoker	38 (82.6%)	86 (86.0%)	0.595
Never‐smoker	8 (17.4%)	14 (14.0%)	
Pathologic tumor size, cm			
<3	17 (37.0%)	49 (49.0%)	0.174
≥3	29 (73.0%)	51 (51.0%)	
Histology			
Pure	29 (73.0%)	62 (62.0%)	0.904
Combine	17 (37.0%)	38 (38.0%)	
SCLC + large cell carcinoma	7 (15.2%)	16 (16.0%)	
SCLC + squamous carcinoma	4 (8.7%)	12 (14.0%)	
SCLC + adenocarcinoma	3 (6.5%)	7 (7.0%)	
Others	3 (6.5%)	3 (3.0%)	
Preoperative biopsy			
No	18 (39.1%)	36 (36.0%)	0.716
Yes	28 (60.9%)	64 (64.0%)	
SCLC	10 (21.7%)	15 (15.0%)	
Other types of cancer	19 (41.3%)	30 (30.0%)	
Others	9 (19.6%)	19 (19.0%)	
Type of resection			
Lobectomy	44 (96.0%)	97 (97.0%)	0.677
Sublobar	2 (4.0%)	3 (3.0%)	
Adjuvant chemotherapy			
No	5 (10.9%)	11 (11.0%)	0.981
Yes	41 (89.1%)	89 (89.0%)	
PET			
No	20 (43.5%)	37 (37.0%)	0.456
Yes	26 (56.5%)	63 (63.0%)	

PCI, prophylactic cranial irradiation; PET, positron emission tomography; SCLC, small cell lung cancer.

## Cerebral recurrence

**Figure 1:**
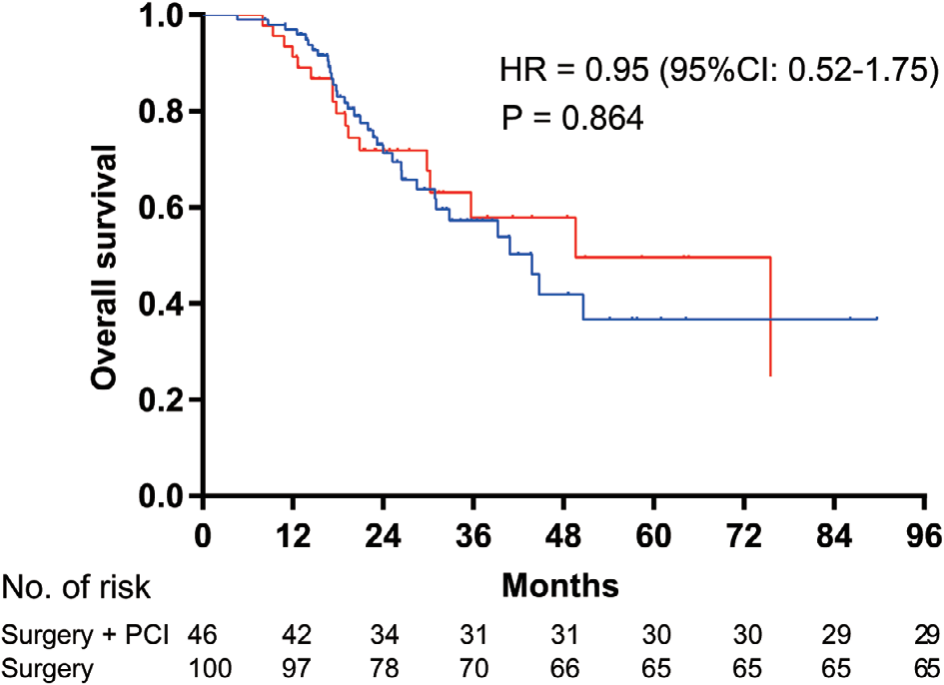
Kaplan‐Meier survival curves for overall survival analysis between the prophylactic cranial irradiation (PCI) after surgery group and the surgery alone group. HR, hazard ratio; CI, confidence interval. 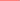 Surgery + PCI, 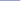 Surgery. HR = 0.95 (95%CI: 0.52–1.75), *P* = 0.864.

Brain metastases developed during the observation window in 12% (12/100) of the cases who did not receive PCI therapy and 10.9% (5/46) of the patients who did receive PCI. Among the 12 patients in the non‐PCI subgroup who developed brain metastases, nine patients received whole brain radiotherapy, three patients received stereotactic radiation. Among the five patients in the PCI subgroup who developed brain metastases, two patients received stereotactic radiation. The incidence of brain metastasis two years after surgery was 10.9% (5/46) and 10.0% (10/100) for PCI and non‐PCI cohorts, respectively. No significant differences were observed with regard to the time to recurrence (*P* = 0.798) (Fig 1).

## Overall and disease‐free survival

**Figure 2:**
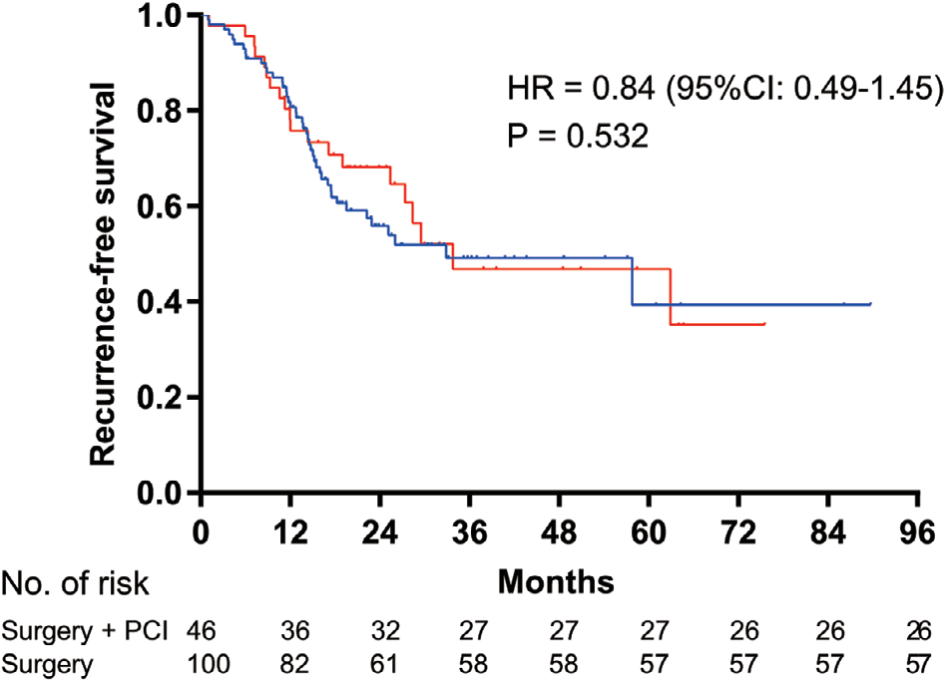
Kaplan‐Meier survival curves for recurrence‐free survival analysis between the prophylactic cranial irradiation (PCI) after surgery group and the surgery alone group. HR, hazard ratio; CI, confidence interval. 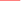
Surgery + PCI, 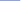
Surgery. HR = 0.84 (95%CI: 0.49–1.45), *P* = 0.532.

**Figure 3:**
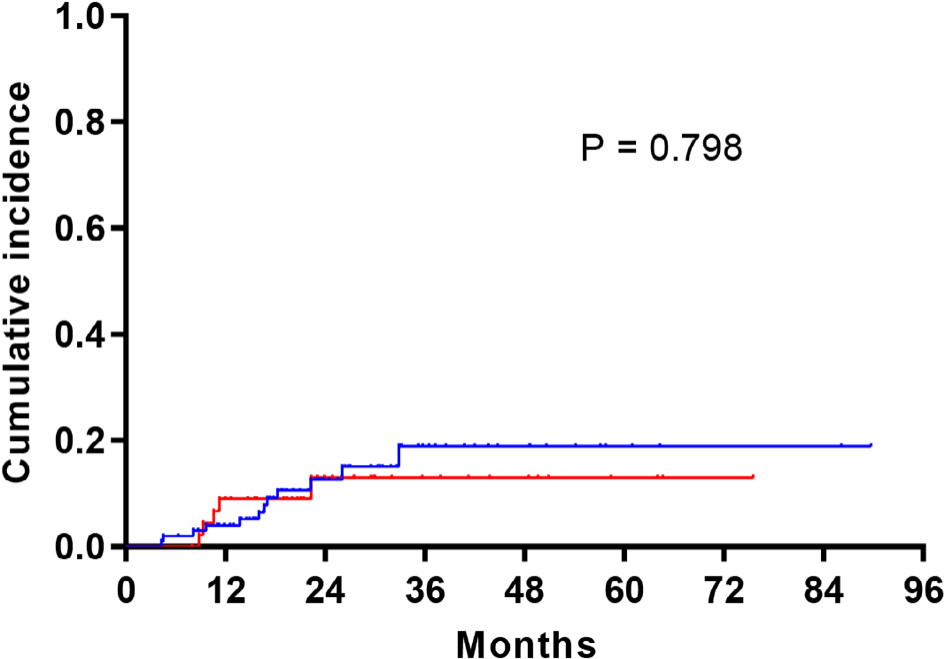
Cumulative incidence of brain metastases in the prophylactic cranial irradiation (PCI) after surgery group and the surgery alone group. 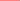
Surgery + PCI, 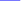
Surgery. *P* = 0.798.

There were a total of 73.9% (34/46) and 78.0% (78/100) of patients with two‐year survival rates in the PCI and non‐PCI cohorts, respectively. PCI therapy did not deliver a significant change in OS (HR = 0.95, 95% CI: 0.52–1.75, *P* = 0.864) (Fig 2). Additionally, there was no significant difference in the RFS (HR = 0.84, 95% CI: 0.49–1.45, *P* = 0.532) in the PCI‐treated group compared with the non‐PCI‐treated group (Fig 3). The median RFS and OS for the groups that received PCI and those who did not receive PCI have not been determined. A multivariate Cox proportional hazards model (Table 2) showed pathologic tumor size as an important predictor of OS. The HR of OS for ≥ 3 cm versus < 3 cm was 1.85 (95% CI: 1.01–3.85; *P* = 0.047).

**Table 2:** Multivariate Cox regression analysis of factors associated with RFS and OS

	RFS		OS	
Characteristics	HR (95% CI)	*P*‐value	HR (95% CI)	*P*‐value
Age				
<65	1		1	
≥65	1.25 (0.73–2.15)	0.417	1.70 (0.93–3.12)	0.086
Gender				
Female	1			
Male	1.14 (0.28–4.60)	0.853	2.79 (0.65–12.03)	0.169
Smoking status				
Smoker	1		1	
Never‐smoker	0.97 (0.66–1.42)	0.875	0.71 (0.18–2.79)	0.625
Pathologic tumor size, cm				
<3	1		1	
≥3	1.42 (0.84–2.40)	0.192	1.85 (1.01–3.85)	0.047
Histology				
Pure	1		1	
Combine	0.83 (0.50–1.39)	0.483	1.09 (0.62–1.92)	0.774
Adjuvant chemotherapy				
No	1		1	
Yes	0.98 (0.43–2.25)	0.969	0.91 (0.38–2.18)	0.823
PET				
No	1		1	
Yes	0.83 (0.49–1.39)	0.476	0.97 (0.53–1.77)	0.916
PCI				
No	1		1	
Yes	0.84 (0.49–1.45)	0.532	0.95 (0.52–1.75)	0.864

CI, confidence interval; HR, hazard ratio; OS, overall survival; PCI, prophylactic cranial irradiation; PET, positron emission tomography; RFS, recurrence‐free survival.

## Discussion

We analyzed the clinical data of p‐T1‐2N0 SCLC and the results demonstrated that postoperative PCI does not reduce the risk of cerebral recurrence (*P* = 0.798). Additionally, PCI failed to increase the OS (HR = 0.84, 95% CI: 0.49–1.45, *P* = 0.532) as well as the DFS (HR = 0.95, 95% CI: 0.52–1.75, *P* = 0.864). This analysis included the largest sample size focused on evaluating PCI in completely resected SCLC with no lymph node involvement. However, the results did not support using PCI in such a population.

Previously, surgery was not considered to be a standard therapy for SCLC. An inquiry into the National Cancer Data Base (NCDB) demonstrated that surgery in cases of SCLC increased from 14.9% to 28.5% between 2004 and 2013.^13^ Such an increase might be explained in part by the development of low dose CT screening and PET scans. Increasingly, SCLC patients are diagnosed at a resectable stage. Furthermore, insufficient diagnostic information from preoperative small biopsies and histologies of NSCLC combined SCLCs were also reasons for surgery as the primary treatment. In a previous study where surgery was utilized as the standard treatment, nearly half of the participants had not been preoperatively diagnosed with SCLC.^14^ In our present study, 92 (63.0%) patients underwent preoperative biopsies. Only 25 (17.1%) patients were preoperatively diagnosed with SCLC before surgery, while 49 (33.6%) patients were diagnosed with other forms of cancer (including large cell carcinoma, squamous carcinoma, adenocarcinoma, as well as unclassified carcinoma).

SCLC is characterized by a rapid multiplying time and elevated risk of developing brain metastases. According to a previous report, the probability of brain metastases reached up to 50% two years following diagnosis.^4^ Clinical data before 2014 in our institution showed that 19.5% of surgically resected SCLC patients suffered brain metastasis during the observation period.^7^ Additionally, the occurrence of brain metastases during p‐stage II and III disease was 28.2% (11/39) and 29.1% (16/55), respectively.^15^ Of the 146 p‐T1‐2N0M0 SCLC patients evaluated in the present study, 63 (43.2%) patients suffered from recurrence at a median of 27.5 months. A total of 17 (11.6%) patients developed brain metastases, which was lower than that of p‐N1‐2M0 patients in previous reports. These results are similar to other reports which demonstrated a risk of brain metastasis in p‐T1‐2N0M0 SCLC patients. Gong *et al*. described the occurrence of brain metastases during p‐stage I disease at 6.25% (2/32).^15^ The NCCN guidelines endorse concurrent chemotherapy and PCI for such patients based on evidence in limited stage SCLC patients.^8^ However, the use of PCI in p‐T1‐2N0M0 SCLC is continually questioned based on the evidence of a comparatively low risk of brain relapse.

In this analysis, the frequency of brain metastases did not show any significant differences between the cohort that did not receive PCI (10.9%) and the PCI‐treated cohort (12.0%). Nor was there an alteration in the risk of cerebral metastases when comparing the groups in regards to the time to recurrence (*P* = 0.798). Similarly, Zhu *et al*. reported the actual risk of brain metastases after three years hovered around 9.7% while PCI did not affect the survival rate during p‐stage I disease.^16^ The current retrospective study shows the incidence of cerebral recurrence two years after surgery reached 10.9% (5/46) and 10.0% (10/100) for PCI and non‐PCI cohorts, respectively. Earlier evidence pointed to a survival benefit to PCI in surgically resected stage I SCLC, including clinical data of pT1‐2N0M0 SCLC from the NCDB which demonstrated that PCI following adjuvant chemotherapy was associated with increased survivals in comparison with no concomitant therapies (HR, 0.52; 95% CI, 0.36–0.75).^17^ This study supports the NCCN recommendations on PCI; however, it should be noted that in the report, OS was not different between the PCI‐post‐surgery group and the group that received just surgery (HR, 1.46; 95% CI, 0.46–4.64).^17^ A multicenter retrospective study from Japan evaluated surgery and PCI in 156 SCLC cases (66 IA/29 IB cases) and multivariate analyses revealed increased OS in patients who received PCI.^18^ However, only 13 of the 156 patients in that study received PCI. Additionally, 61 of the 156 patients were not p‐stage I (17 IIA, six IIB, 31 IIIA, three IIIB, three IV) and the results could not support PCI for such p‐stage I patients.^18^

The retrospective design of the current study should be considered while interpreting its conclusions. PET as standard care presurgery may increase survival. In this study, 57 (39.0%) of 146 participants did not receive PET before preoperatively which may have introduced bias. Furthermore, strategies to effectively prevent neurocognitive degeneration after PCI are not certain. These aspects significantly influence treatment management and need to be assessed in prospective studies.

In conclusion, the current retrospective analysis suggests that PCI is perhaps unnecessary in cases of resected p‐T1‐2N0M0. Revised versions of both national and international guidelines should readdress this matter.

## Disclosure

The authors declare no conflicts of interest.

## Supporting information


**Table S1.** Failure pattern of the PCI and non‐PCI cohorts
