# Supplementary material for: Does surgically resected small‐cell lung cancer without lymph node involvement benefit from prophylactic cranial irradiation?
Source: Thorac Cancer. 2020 Mar 6;11(5):1239–44. doi: 10.1111/1759-7714.13381 (PMC7180625; doi:10.1111/1759-7714.13381)
Supplement: Supplementary file 1 — Table S1. Failure pattern of the PCI and non‐PCI cohorts [file TCA-11-1239-s001.doc]

**Supplemental Table 1.** Failure pattern of the PCI and non-PCI cohorts

| PCI therapy | (n = 20) | Non PCI therapy | (n = 43) |
| --- | --- | --- | --- |
| Mediastina and lung | 9 | Mediastina and lung | 14 |
| Mediastina and lung + brain | 4 | Mediastina and lung + brain | 5 |
| Mediastina and lung + brain + other distant metastasis | 1 | Mediastina and lung + brain + other distant metastasis | 6 |
| Mediastina and lung+ other distant metastasis | 5 | Mediastina and lung + other distant metastasis | 13 |
| Brain only | 0 | Brain only | 1 |
| Others | 1 | Others | 4 |
